# Analysis of the influence factors of cervical lymph node metastasis in Papillary thyroid carcinoma: A retrospective observational study

**DOI:** 10.1097/MD.0000000000035045

**Published:** 2023-09-08

**Authors:** Jinfeng Lou, Jiahui Yang, Yong Luo, Ye Zhu, Zheng Xu, Tebo Hua

**Affiliations:** a Department of Anesthesiology, Ningbo Medical Centre Lihuili Hospital, Ningbo, Zhejiang, P.R. China; b Department of Thyroid Breast Surgery, Ningbo Medical Centre Lihuili Hospital, Ningbo, Zhejiang, P.R. China.

**Keywords:** cervical lymph node metastasis, neck lymph node dissection, surgery, Papillary thyroid carcinoma, thyroid cancer

## Abstract

Papillary thyroid carcinoma (PTC) is the most common type of thyroid cancer, and surgery is crucial for curing PTC. PTC patients often experience lymph node metastasis (LNM) in the neck, and central lymph node metastasis (CLNM) significantly affects the recurrence rate of PTC. Therefore, the thoroughness of the surgery is particularly important for the treatment of PTC. However, there is still controversy regarding the choice of surgical approach. This study retrospectively analyzed the clinical data of 69 PTC patients treated at our hospital from December 2019 to April 2022 and clinically analyzed the high-risk factors for neck LNM. In this study, the patients aged ≤ 55 years were examined in which the number of patients with CLNM were 42 cases (80.77%), tumor diameter >2 cm were 15 cases (100%), the multifocal carcinoma were 38 cases (88.37%) and the involvement of membrane were 38 cases (80.85%), the number of patients whose had lateral cervical lymph node metastasis (LLNM), respectively 43 cases (82.69%), 14 cases (93.33%), 39 cases (90.7%) and 40 cases (85.11%),all of these factors were associated with cervical LNM (*P* < .05), but was not correlation with sex, double lobe carcinoma, extra glandular invasion and hashimoto (*P* > .05). The patient’s age and number of cancers were independent risk factors for LNM in the central region of the neck (*P* < .05), while the patient’s age, tumor size and number of cancers were significant risk factors for LNM in the lateral cervical region (*P* < .05). We concluded that cervical LNM was related with the high-risk factors of patient’s age, tumor size, multifocal carcinoma in PTC. Especially, modified radical cervical dissection or selective cervical dissection was suggested in the PTC patients who were younger than 42.5 years old, with tumor diameter larger than 2 cm and multifocal carcinoma.

## 1. Introduction

Thyroid carcinoma (TC) is one of the most common endocrine malignancies, and its occurrence has been increasing in recent years. Papillary thyroid carcinoma (PTC) is the most common type of TC, accounting for about 70% to 80% of cases, which significantly affects people’s health and quality of life.^[1–4]^ PTC is characterized by high differentiation, low malignancy, and slow growth. The clinical prognosis of most PTC patients is within the range of 5-year and 10-year survival rates of 95% and 90%, respectively.^[5]^ However, it is worth noticing that PTC patients often have lymph node metastasis (LNM) in the neck, with a metastasis rate of 20% to 50%.^[6–8]^ In addition, central lymph node metastasis (CLNM) has a significant impact on the recurrence rate of PTC.^[9]^ Therefore, the thoroughness of surgery is particularly important for the treatment of PTC. However, the choice of surgical approach is still controversial, especially regarding whether to perform lateral neck lymph node dissection in PTC patients with clinically negative lymph nodes (cN0 stage).

In this study, we retrospectively analyzed the clinical data of 69 PTC patients who were diagnosed and treated in our hospital from December 2019 to April 2022, and conducted a clinical analysis of the high-risk factors for cervical LNM, to provide a theoretical basis for whether PTC patients should undergo cervical lymph node dissection.

## 2. Patients and methods

### 2.1. Patients and clinical data

We retrospectively analyzed the clinical data of 69 patients who were diagnosed with PTC and treated in the Department of Thyroid Breast Surgery in our hospital from December 2019 to April 2022. Inclusion criteria: Postoperative pathological diagnosis of PTC; No history of neck surgery, family history, or radiation exposure before surgery; All patients underwent ipsilateral central lymph node dissection and lateral cervical lymph node dissection (unilateral); Complete patient data were available. Of these patients, 19 were male and 50 were female, aged between 21 and 71 years, with an average age of 44.55 ± 13.127 years. All patients underwent neck ultrasound (US) and enhanced CT before surgery. The clinical stage of thyroid cancer and postoperative LNM were calculated based on the AJCC eighth edition (2015) TNM staging criteria for thyroid cancer. Clinical and pathological data are detailed in Table 1. Under the Declaration of Helsinki, this study involving human participants were reviewed and approved by the Ethics Committee of Ningbo Medical Centre Lihuili Hospital employee (Approval NO.KY2022PJ236). Written informed consent for participation was not required for this study in accordance with the national legislation and the institutional requirements.

**Table 1:** Univariate analysis of cervical lymph node metastasis in 69 cases of PTC.

Clinical and pathological features		N = 69	Central lymph nodes[n(%)]	*χ^2^*	*P* value	Lateral cervical lymph nodes[n(%)]	*χ^2^*	*P* value
			n = 50			n = 51		
Gender	Male	19	14 (73.68%)	0.02	.889	16 (84.21%)	1.442	.23
	Female	50	36 (72.0%)			35 (70%)		
Age (yr)	>55	17	8 (47.06%)	7.296	.007	8 (47.06%)	8.437	.004
	≤55	52	42 (80.77%)			43 (82.69%)		
Tumor size (cm)	<1	17	7 (41.18%)	16.206	.001	7 (41.18%)	18.602	.000
	1–2	21	14 (66.67%)			14 (66.67%)		
	2–4	15	15 (100%)			14 (93.33%)		
	≥4	16	14 (87.5%)			16 (100%)		
Bilateral cancer	Yes	22	15 (68.18%)	0.297	.586	16 (72.73%)	0.024	.878
	No	47	35 (74.47%)			35 (74.47%)		
Multifocal cancer	Yes	43	38 (88.37%)	14.473	.000	39 (90.7%)	16.673	.000
	No	26	12 (46.15%)			12 (46.15%)		
Hashimoto thyroiditis	Yes	12	11 (91.67%)	2.685	.101	11 (91.67%)	2.375	.123
	No	57	39 (68.42%)			40 (74.18%)		
Capsular invasion	Yes	47	38 (80.85%)	5.197	.023	40 (85.11%)	9.578	.002
	No	22	12 (54.55%)			11 (50%)		
Extrathyroidal extension	Yes	31	25 (80.65%)	1.888	.169	26 (83.87%)	2.895	.089
	No	38	25 (65.79%)			25 (65.79%)		
Pathological stage	I	38	30 (78.95%)	8.114	.044	32 (84.21%)	15.136	.002
	II	1	0			0		
	III	6	2 (33.33%)			1 (17%)		
	IV	24	18 (75%)			18 (75%)		

PTC = Papillary thyroid carcinoma.

### 2.2. Surgery strategies

All patients underwent standardized surgical treatment under general anesthesia with endotracheal intubation. The surgical procedure involved bilateral thyroidectomy and ipsilateral central lymph node dissection and lateral cervical lymph node dissection (unilateral). The central lymph node dissection included the lymph nodes below the hyoid bone, above the innominate vein, inside the carotid sheath, behind the prevertebral fascia, and included the lymph nodes around the trachea, anterior to the trachea, in front of the larynx, and behind the recurrent laryngeal nerve. The lateral cervical lymph node dissection included the lymph nodes below the hypoglossal nerve, above the subclavian vein, in front of the sternocleidomastoid muscle, and behind the prevertebral fascia, and included lymph nodes in the II to V regions of the cervical. Excised tissues were sent for pathological diagnosis, and 2 pathologists reviewed the slides together.

### 2.3. Statistical analysis

SPSS 21.0 statistical software package was used for statistical analysis of the data. The chi-square test was used for count data, and multiple logistic regression analysis was used for multivariate analysis. Receiver operating characteristic (ROC) curves were drawn, and the Youden index was calculated. The cutoff value corresponding to the maximum Youden index was used as the best cutoff value for the scoring system. *P* < .05 was considered statistically significant.

## 3. Results

### 3.1. Univariate analysis of high-risk factors for LNM in PTC

Among the 69 patients, 50 had CLNM and 51 had lateral cervical lymph node metastasis (LLNM). Age, tumor diameter, multifocal carcinoma, capsule invasion, and tumor pathological stage were all significantly correlated with CLNM and LLNM (*P* < .05), while gender, bilateral carcinoma, extrathyroidal invasion, and Hashimoto thyroiditis were not significantly correlated with CLNM and LLNM (*P* > .05), as shown in Table 1.

### 3.2. Multivariate analysis of high-risk factors for PTC cervical LNM

Multivariate logistic regression analysis showed that patient age and number of cancer foci were independent risk factors for central cervical LNM (*P* < .05), while gender, tumor size, capsule involvement, and extrathyroidal extension were not correlated with central cervical LNM (*P* > .05), as shown in Table 2. Patient age, tumor size, and number of cancer foci were independent risk factors for LLNM (*P* < .05), while gender, capsule involvement, and extrathyroidal extension were not correlated with LLNM (*P* > .05), as shown in Table 3.

**Table 2:** Multivariate analysis of 69 PTC cases with central cervical lymph node metastasis.

Factor	Partial regression coefficient B	Standard error S.E.	Wald value *χ^2^*	95%CI	*P* value	Exp (B)
Gender	0.831	0.885	0.883	0.405–13.008	.347	2.297
Age	1.578	0.803	3.864	1.005–23.365	.049	4.845
Tumor size	−0.744	0.407	3.3397	0.214–1.055	.068	0.475
Number of cancer foci	−1.809	0.750	5.817	0.038–0.713	.016	0.164
Invasion of the capsule	−1.151	0.911	1.597	0.053–1.886	.206	0.316
Extrathyroidal extension	0.285	1.043	0.075	0.172–10.267	.785	1.330

PTC = Papillary thyroid carcinoma.

**Table 3:** Multivariate analysis of risk factors for lateral cervical lymph node.

Factor	Partial regression coefficient B	Standard error S.E.	Wald value *χ^2^*	95%CI	*P* value	Exp (B)
Gender	−0.384	1.104	0.121	0.078–5.930	.728	0.681
Age	1.930	0.977	3.905	1.016–46.711	.048	6.888
Tumor size	−1.219	0.565	4.648	0.098–0.895	.031	0.296
Number of cancer foci	−2.084	0.862	5.841	0.023–0.674	.016	0.124
Invasion of the capsule	−2.145	1.130	3.605	0.013–1.072	.058	1.117
Extrathyroidal extension	0.907	1.387	0.427	0.163–37.507	.513	2.476

### 3.3. ROC curve analysis of LNM in the lateral cervical region

**Figure 1.**
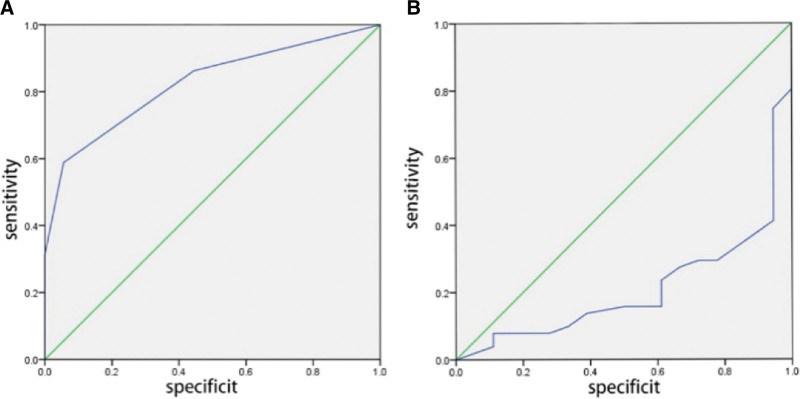
(A) ROC curve analysis of tumor size predicting lateral cervical lymph node metastasis in PTC patients. (B) ROC curve analysis of age predicting lateral cervical lymph node metastasis in PTC patients. , ROC = receiver operating characteristic.

Multivariate logistic regression analysis showed that tumor size and age were correlated with LNM in the lateral cervical region of PTC patients, which could be used as predictive indicators for lateral cervical metastasis. To further investigate the relationship between tumor size, age, and LLNM in PTC patients, we constructed ROC curves for 69 PTC patients to determine the critical values for these factors in predicting LLNM. As shown in Figure 1A, the critical value for tumor size was 2.0 cm (sensitivity = 0.569, specificity = 0.944, an area under the curve (AUC) = 0.798, *P* < .001). As shown in Figure 1B, the critical value for age was 42.5 years (sensitivity = 0.412, specificity = 0.056, AUC = 0.209, *P* < .001).

## 4. Discussion

Currently, surgical treatment is the main approach for PTC, and the choice of surgical approach is crucial. For patients who are found to have CLNM or LLNM on preoperative US or physical examination, radical cervical lymph node dissection is undoubtedly necessary. However, it has been a clinical controversy about which cN0 PTC patients need to undergo lateral cervical lymph node dissection, and there is currently no consensus on the indications for prophylactic lymph node dissection and the scope of dissection remains controversial. Studies have shown that the size, number, and ratio of metastatic lymph nodes may affect local recurrence in PTC patients.^[10]^ Some studies have shown that CLNM (*P* = .010) and LLNM (*P* = .001) were identified as important risk factors for disease-free survival, especially LLNM.^[11]^ Recurrence and reoperation can cause great pain and economic burden to patients, and affect their disease-free survival. However, blindly performing radical cervical dissection for patients without suspicious LNM before surgery can cause great physical trauma, economic losses, and psychological pressure. Moreover, the prognosis of differentiated thyroid cancer is better, and performing a second surgery after discovering suspicious lymph nodes may not shorten the patient’s overall survival time, but currently, there is a lack of multi-center prospective studies to confirm this view. Therefore, preoperative assessment of high-risk factors in PTC patients is crucial for individualized treatment and avoiding unnecessary cervical lymph node dissection.

Due to the limitations of preoperative US or CT examination, some patients with occult or small metastatic cancer may have false negative cervical lymph node results, so it cannot be blindly assumed that cervical lymph node dissection can be omitted for cN0 PTC patients. Some scholars believed that patients with capsular invasion are significantly more likely to have LLNM than those without capsular invasion. Therefore, cervical lymph node dissection in the III and IV regions is important for cN0 stage thyroid cancer patients at high risk in the cervical, especially for patients with VI lymph node positivity and thyroid capsule invasion. It can timely detect and remove hidden metastasis in the lateral cervical lymph nodes, which can extend the patient’s survival time to some extent. He also believes that for young patients, once III and IV regional LNM is detected, cervical lymph node dissection should be completed, at least II-IV regional lymph node dissection should be performed.

This study showed that among 69 patients with PTC, 50 had CLNM and 51 had LLNM. Age, tumor size, and multifocality were all correlated with cervical LNM (*P* < .05), and multivariate logistic regression analysis showed that age and number of cancer foci were independent risk factors for CLNM (*P* < .05), while age, number of cancer foci, and tumor size were independent risk factors for LLNM (*P* < .05). Other studies have also identified age, tumor size, and multifocality as risk factors for CLNM, with multifocal PTC having a higher rate of CLNM than single-focal tumors.^[12,13]^ Feng JW et al showed that 24.7% of patients had multifocal PTC, and it was found that the frequency of CLNM was higher in the multifocal group, which inferred that the high frequency of LNM occurred in multifocal PTC, the increase in tumor number is associated with an increase in risk and is a high-risk factor for CLNM and LLNM, suggesting that the number of tumor lesions is more important than tumor location, consistent with the results of this study.^[14]^ Chen Y et al showed that the incidence of CLNM was related to male, age < 55 years, the increase in tumor size is closely related, and the increase in tumor size and incidence of CLNM makes patients extremely susceptible to LLNM.^[15]^ Additionally, other studies have identified risk factors for CLNM in PTC patients, including male gender, young age, and increasing tumor size.^[16,17]^ Therefore, it can be inferred that prophylactic lateral cervical lymph node dissection should be performed for patients with these high-risk factors. This study also showed that the critical value for age in predicting LLNM was 42.5 years (sensitivity = 0.412, specificity = 0.056, AUC = 0.209, *P* < .001), and the critical value for tumor size was 2.0 cm (sensitivity = 0.569, specificity = 0.944, AUC = 0.798, *P* < .001) as determined by ROC curve analysis. Thus, patients younger than 42.5 years and those with multiple cancer foci, tumor diameter >2.0 cm, and invasion of the capsule and surrounding tissues warrant special attention. Although no prospective studies have been conducted to demonstrate whether routine prophylactic cervical lymph node dissection can improve prognosis, we have found that these factors are poor prognostic factors for PTC patients.

There is an ongoing debate about whether to perform cervical lymph node dissection in cN0 PTC patients, and there is currently no consensus on which cN0 patients need cervical lymph node dissection. This study believes that central lymph node dissection is necessary for cN0 PTC patients and should be routinely performed. For patients with high-risk factors before surgery (such as age, multiple tumors, tumor size), selective lateral cervical dissection or modified radical cervical dissection can be added. This approach avoids overtreatment, detects and removes hidden lateral cervical lymph node metastases in a timely manner, reduces the rate of reoperation, and improves the quality of life of patients. In addition, for patients younger than 42.5 years, with multiple tumors, tumor diameter larger than 2.0 cm, and invasion of the capsule and surrounding tissue, lateral cervical lymph node dissection is recommended. In summary, whether or not to perform cervical lymph node dissection in cN0 PTC patients should be based on individualized treatment and avoid unnecessary preventive lymph node dissection.

## 5. Conclusion

Cervical LNM was related to the high-risk factors of the patient’s age, tumor size, and multifocal carcinoma in PTC. Especially, modified radical cervical dissection or selective cervical dissection was suggested in the PTC patients who’s younger than 42.5 years old, with tumor diameter larger than 2cm and multifocal carcinoma.

## Author contributions

**Data curation:** Jinfeng Lou, Zheng Xu, Tebo Hua.

**Funding acquisition:** Tebo Hua.

**Investigation:** Jinfeng Lou, Zhu Ye.

**Methodology:** Jinfeng Lou, Tebo Hua.

**Resources:** Jiahui Yang, Luo Yong.

**Validation:** Jiahui Yang.

**Writing – original draft:** Jinfeng Lou.

**Writing – review & editing:** Tebo Hua.
